# Flex Meta-Storms elucidates the microbiome local beta-diversity under specific phenotypes

**DOI:** 10.1093/bioinformatics/btad148

**Published:** 2023-03-22

**Authors:** Mingqian Zhang, Wenke Zhang, Yuzhu Chen, Jin Zhao, Shunyao Wu, Xiaoquan Su

**Affiliations:** College of Computer Science and Technology, Qingdao University, Qingdao, China; College of Computer Science and Technology, Qingdao University, Qingdao, China; College of Computer Science and Technology, Qingdao University, Qingdao, China; College of Computer Science and Technology, Qingdao University, Qingdao, China; College of Computer Science and Technology, Qingdao University, Qingdao, China; College of Computer Science and Technology, Qingdao University, Qingdao, China

## Abstract

**Motivation:**

Beta-diversity quantitatively measures the difference among microbial communities thus enlightening the association between microbiome composition and environment properties or host phenotypes. The beta-diversity analysis mainly relies on distances among microbiomes that are calculated by all microbial features. However, in some cases, only a small fraction of members in a community plays crucial roles. Such a tiny proportion is insufficient to alter the overall distance, which is always missed by end-to-end comparison. On the other hand, beta-diversity pattern can also be interfered due to the data sparsity when only focusing on nonabundant microbes.

**Results:**

Here, we develop Flex Meta-Storms (FMS) distance algorithm that implements the “local alignment” of microbiomes for the first time. Using a flexible extraction that considers the weighted phylogenetic and functional relations of microbes, FMS produces a normalized phylogenetic distance among members of interest for microbiome pairs. We demonstrated the advantage of FMS in detecting the subtle variations of microbiomes among different states using artificial and real datasets, which were neglected by regular distance metrics. Therefore, FMS effectively discriminates microbiomes with higher sensitivity and flexibility, thus contributing to in-depth comprehension of microbe–host interactions, as well as promoting the utilization of microbiome data such as disease screening and prediction.

**Availability and implementation:**

FMS is implemented in C++, and the source code is released at https://github.com/qdu-bioinfo/flex-meta-storms.

## 1 Introduction

Microbes exist in various ecosystems in the form of microbiome, and they have a close interaction with their habitats. At present, studies on microbiome mainly rely on bioinformatical analysis of sequencing data, which can quickly decode the structures and functions of microbiomes, thus explain their roles in human health or environment (Thompson et al. 2017; Proctor et al. 2019). Beta-diversity is a key foundation for microbiome researches and applications, for it builds the connection between complex characteristics of microbial communities and their phenotype information (Knight et al. 2018).

Currently, beta-diversity is measured in a “global alignment” mode, which calculates distances between microbiomes by all microbial members within the communities. The widely used distance metrics are roughly in two categories. One is vector or statistical distance, like Bray–Curtis distance, Jessen–Shannon divergency, Jaccard distance, etc. Such methods emphasize the overlapped community members but ignored their relationships, which can lead to the deviation of beta-diversity patterns (Sun et al. 2022); the other is phylogeny-based dissimilarities, e.g. UniFrac algorithm (Catherine and Rob 2005; Hamady et al. 2010; McDonald et al. 2018), Meta-Storms series algorithm (Su et al. 2012, 2013; Jing et al. 2019), Phylo-RPCA (Cameron et al. 2022), etc. making the distances more comprehensive by considering microbes’ evolution (Matteo et al. 2020). Both approaches employ all microbial features, however, may miss subtle changes among microbiomes under specific cases. For example, only a small part of microbes is associated with autism spectrum disorder (Son et al. 2015), which are not sufficient to affect the “whole-community-level” distance, thereby obscuring the understanding on microbe–disease association.

Meanwhile, microbiome beta-diversity has also been widely exploited in status identification and classification. Usually, statistical approaches (e.g. LefSe (Nicola et al. 2011)) or regular machine learning (Goecks et al. 2020) (e.g. random forest, etc.) can find out the key taxa as bio-markers, based on which we can build models or indices to infer the phenotype of the host or habitat (Su et al. 2020). Since the resolution of microbiome profiling has been largely improved (Ye et al. 2019; Meyer et al. 2022), models or indices are often built with detailed features like species or ASVs (amplicon sequence variant) for high specificity. On the other side, due to sequencing errors and algorithm inaccuracy of short reads (Edgar 2017), it is possible that microbiomes only share few markers. Such data sparsity can also significantly interfere the beta-diversity patterns, as well as lead to erroneous results in status prediction.

## 2 Results

### 2.1 Interpretation and algorithm design of microbiome local alignment

Based on the preliminary concept of microbiome “local alignment” that we previous proposed (Su 2021), here, we concretize a “local alignment” distance algorithm named Flex Meta-Storms (FMS), and implement it as software packages. Using a flexible member extraction, FMS captures a subset of interest from complex communities and calculates a normalized phylogenetic distance between sample pair. Such effects elucidate the beta-diversity with balanced sensitivity and accuracy than existing distance metrics, especially for phenotypes like human diseases.

**Figure 1.**
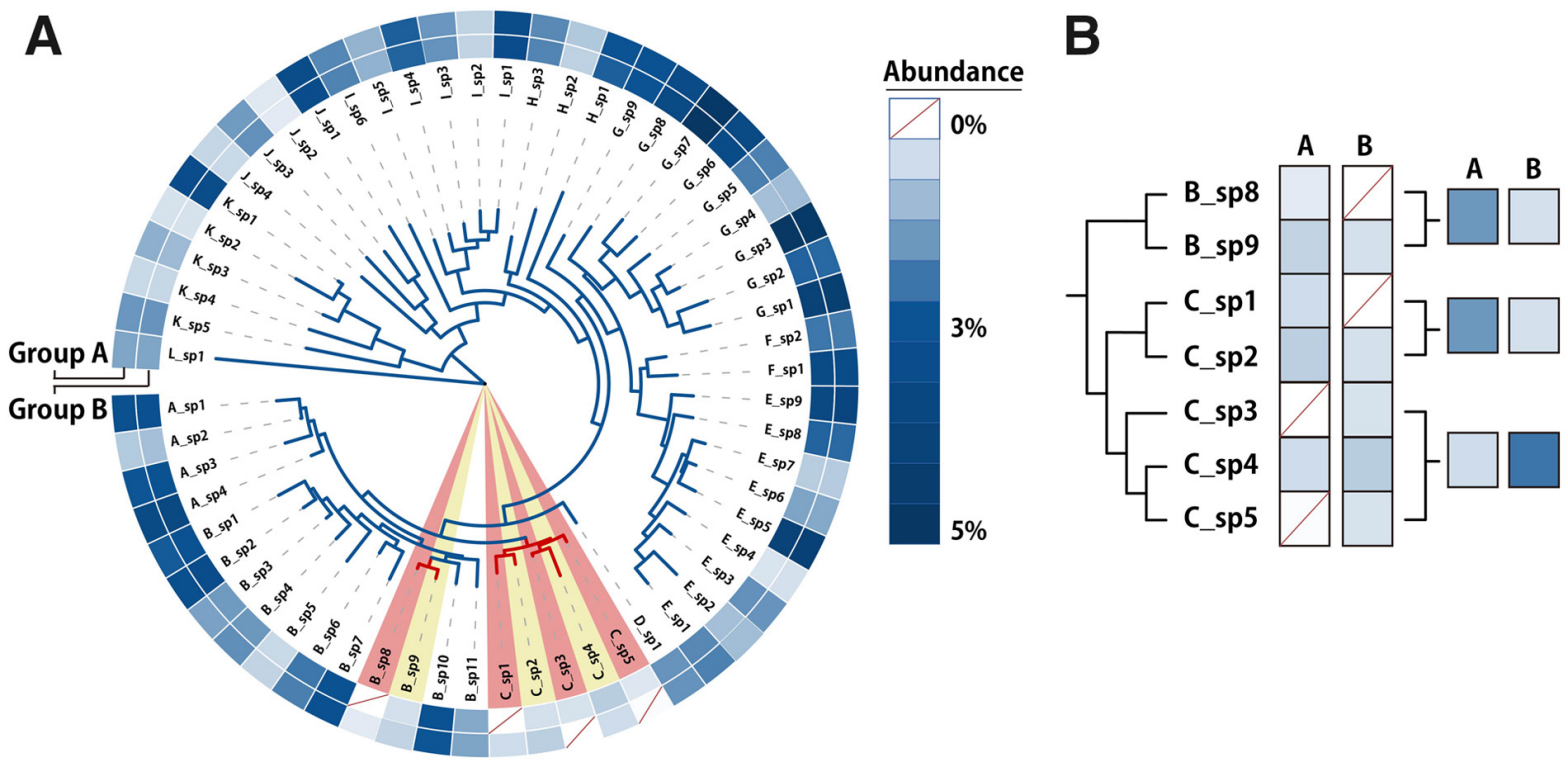
An example of the Flex Meta-Storms algorithm. (A) Phylogenetic tree and species distribution of two microbiome sets. (B) Distribution of target members.

Here, we illustrate the “local alignment” FMS algorithm by an example. As shown in Fig. 1A, Groups A and B are microbiomes in two different statuses. Most members between the two groups are similarly distributed, while variations of their beta-diversity are only related to a small fraction of species with low abundance (i.e. species *B_sp8*, *C_sp1*, *C_sp3*, and *C_sp5*, highlighted in red in Fig. 1A; denoted as *exact markers*). In this case, the distinction of beta-diversity between Groups A and B cannot be adequately reflected when using the “global alignment” methods, consequently interfering diversity pattern recognition between statuses. On the other side, it is possible that microbiomes may share few exact markers due to data sparsity or sequencing/profiling errors. For example, Group B does not have species *B_sp8* and *C_s1*, but contains their close relatives of *B_sp9* and *C_sp2* (Fig. 1A), thus concentrating only on exact markers but ignoring linkages among microbes can also lead to false positive or false negative.

The FMS algorithm focuses on the target members that consist of two parts: (i) exact markers; and (ii) *approximate markers* that have very close phylogeny or metabolic functions to the exact markers. Firstly, exact markers (highlighted in red in Fig. 1A) are detected by bio-marker selection (e.g. statistical tests or machine learning) or assigned manually (e.g. microbes of interest). FMS then flexibly locates approximate markers (e.g. species *B_sp9*, *C_sp2*, and *C_sp4* in Fig. 1A, highlighted in yellow) by phylogeny and functions, and extracts all targeted members from the communities (Fig. 1B). Notably, abundances of approximate markers are not directly employed but are weighted by distances to their nearest exact markers. Finally, FMS calculates the normalized phylogeny-based Meta-Storms distances between sample pairs on target members thus reveals the association between microbiome compositions and status. The detailed procedure is also described in Section 2 and Supplementary Fig. S1.

### 2.2 Assessment of Flex Meta-Storms on artificial datasets

**Figure 2.**
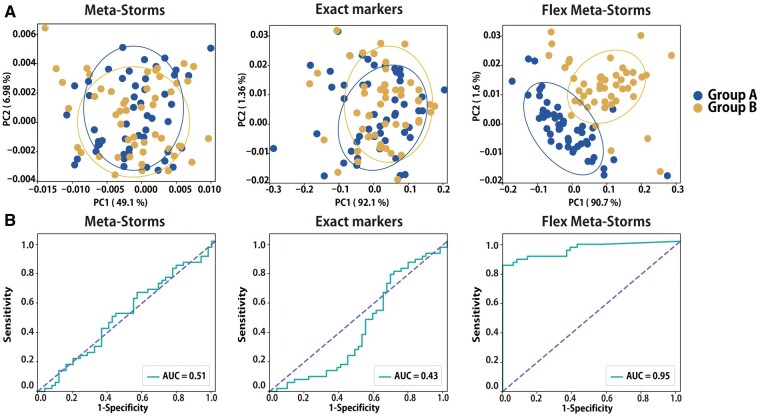
Beta-diversity patterns of the artificial dataset using different distances. (A) PCoA analysis results. (B) ROC of KNN-based status prediction. “Exact markers” denotes the Meta-Storms distance using only exact markers.

The feasibility of FMS was verified by analysis of an artificial dataset (Table 1). This dataset simulated 100 samples of Groups A and B according to the microbial patterns as Fig. 1. We calculated their pairwise distances using the Meta-Storms algorithm by all members (global alignment) and only bio-markers, and the FMS algorithm (local alignment), respectively. As shown in Fig. 2A, the principal coordinate analysis (PCoA) intuitively showed the high sensitivity of FMS in beta-diversity analysis, while others failed in differentiating the two groups. Then, we furtherly predicted the group information by K-nearest neighbors (KNN) (Su et al. 2020) and evaluated the performance of three metrics by leave-one-out tests. The operating characteristic curve (ROC) also exhibited the consistent results as PCoA (Fig. 2B): the FMS obtained the top AUC (area under the ROC) of 0.95 but that of global alignment and biomarkers was only below 0.6.

**Table 1.** Description of datasets.

Dataset	No. of samples	Source	Type	Description
Artificial dataset	100	Simulation	OTU table	Artificial samples of Fig. 1
Real dataset I	88	NCBI PRJNA282013 (Son et al. 2015)	16S amplicon	ASD and healthy control
Real dataset II	104	NCBI PRJNA290926 (Baxter et al. 2016)	16S amplicon	CRC and healthy control

### 2.3 Awareness of the hidden beta-diversity under partial fraction between healthy states

Numerous studies have shown that autism spectrum disorders (ASD) were associated with only a small subset of gut microbes such as *Dialister*, *Lactobacillus*, and *Parabacteroides* (Francesco et al. 2017; Lu et al. 2021; Jiayin et al. 2022). Here, we employed Real dataset I from an ASD study (Table 1) for beta-diversity analysis and compared the performance of global alignment metrics (including Bray–Curtis, Meta-Storms, UniFrac, and Phylo-RPCA) and local alignment of FMS algorithm by PCoA pattern detection, permutational multivariate analysis of variance (PERMANOVA; permutation *n *=* *999), and KNN-based disease classification. To avoid the bias introduced from advanced bio-marker selection strategies, here, we only used Wilcoxon rank-sum test for exact marker selection in FMS (features with *P*-value < 0.01 were selected).

**Figure 3.**
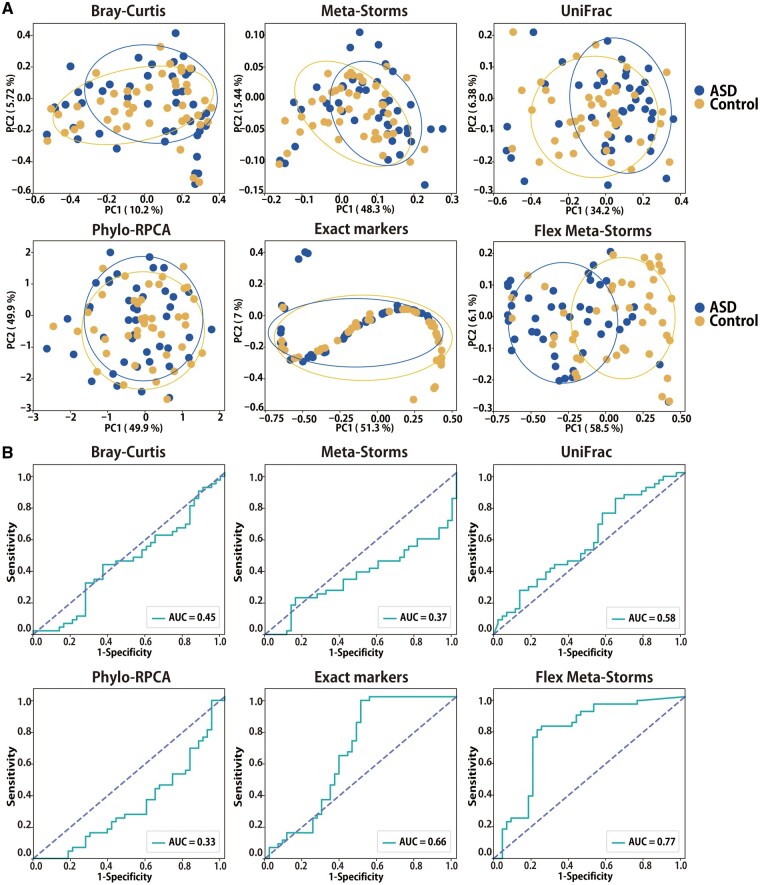
Beta-diversity patterns of real dataset I (ASD) using different distances. (A) PCoA analysis results. (B) ROC of KNN-based status prediction. “Exact markers” denotes the Meta-Storms distance using only exact markers.

As shown in Fig. 3A, we observed that all the global distance metrics were not able to distinguish ASD samples from healthy controls in PCoA patterns, and their *P*-values of PERMANOVA tests were not significant (Table 2A; cutoff was set as 0.01; Supplementary Fig. S2), thus produced weak AUC (<0.6; Fig. 3B) in ASD detection. We noticed that the Meta-Storms distance with only exact markers was sensitive to the ASD (*P*-value < 0.01; AUC = 0.66), but the beta-diversity pattern in PCoA was distorted (“Exact markers” panels in Fig. 3A and B) due to the lack of shared markers among samples (only seven markers that took a proportion of 1.75%). On the other side, with additional 92 approximate markers, the hidden linkage between ASD and the gut microbes was uncovered and reflected by FMS (*P*-value < 0.01; Fig. 3A and Supplementary Fig. S2; Beta-diversity was verified by ANOSIM test in Supplementary Table S1A, and homogeneity was assessed by multivariate dispersion test in Supplementary Table S2A), resulting a reliable disease classification (Fig. 3B; AUC = 0.77). In addition, the ASD samples and healthy controls exhibited homogeneity of dispersion on all distances (*P*-value > 0.01; Supplementary Table S2A).

**Table 2.** Results of PERMANOVA test between different status.^a^

Distance metrices	Bray–Curtis	Meta-Storms	UniFrac	Phylo-RPCA	Exact markers	FMS
(A) Real dataset I						
* R* ^2^	0.01176	0.02106	0.02444	0.00943	0.07132	0.14844
* P*-value	0.375	0.093	0.034	0.457	0.001	0.001
(B) Real dataset II						
* R* ^2^	0.0213	0.04758	0.03066	0.01194	0.25774	0.33849
* P*-value	0.001	0.003	0.012	0.298	0.001	0.001

a “Exact markers” denotes the Meta-Storms distance using only exact markers.

### 2.4 Highlighting the overall beta-diversity associated with phenotypes

 Different from only a subtle fraction of microbes was changed with ASD, more diseases were reported to affect the microbiome at the whole-community level such as colorectal cancer (CRC) (Wirbel et al. 2019). Thus, we repeated the analytical procedures of ASD microbiomes by real dataset II from a CRC study (Table 1), which verified the applicability of FMS algorithm on the dynamics of the majority members among communities.

**Figure 4.**
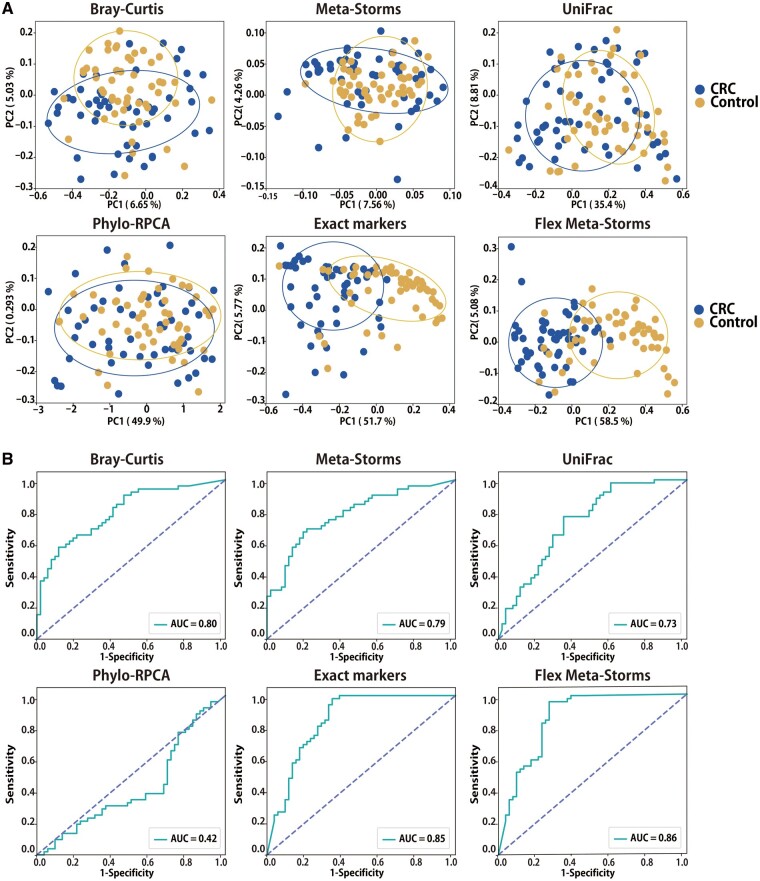
Beta-diversity patterns of real dataset II (CRC) using different distances. (A) PCoA analysis results. (B) ROC of KNN-based status prediction. “Exact markers” denotes the Meta-Storms distance using only exact markers.

As shown in Table 2B, most approaches (except Phylo-RPCA) successfully caught the distinct gut microbial pattern between CRC patients and healthy controls (Fig. 4A; PERMANOVA test *P*-value < 0.01; Supplementary Fig. S3), enabling the disease classification using KNN (Fig. 4B; AUC > 0.7). Here, the PCoA pattern parsed by only biomarkers was largely improved than that of ASD since exact markers were more abundant in CRC samples (average proportion: 10.11% of CRC versus 1.75% of ASD; “Exact marker” panel in Fig. 4A). Among them, the FMS algorithm achieved the highest *R*^2^ of PERMANOVA test (*R*^2^ = 0.34, *P*-value < 0.01; beta-diversity was verified by ANOSIM test in Supplementary Table S1B, and homogeneity was assessed by multivariate dispersion test in Supplementary Table S2B) and AUC of disease classification (AUC = 0.86). This superiority was derived from it rationally amplified the variation between states by the exact and approximate markers flexibly extracted from the whole communities.

## 3 Discussion

As a fundamental characteristic of microbiome, beta-diversity has been used to quantify the difference among communities, thus links the microbial compositions with meta-data. Different from existing approaches that are mainly based on the whole-community-level distances, the Flex Meta-Storms algorithm in local-alignment type focuses on partial members that are flexibly selected, which achieves an optimal sensitivity and specificity in describing the diversity pattern for specific phenotypes. In other words, FMS enhances the discrimination of microbiomes rather than a typical overall beta-diversity distance metric. Coupled with further analytical steps like PCoA, KNN, and PERMANOVA tests, FMS could better leverage its ability to decipher the hidden beta-diversity of microbiomes. Since the local alignment also relies on the “key” exact markers that deduce the target members, the performance of FMS can be furtherly enhanced by state-of-the-art biomarker selection (Nearing et al. 2022), e.g. NetMoss (Xiao et al. 2022), ALDEx2 (Fernandes et al. 2014), ANCOM-II (Mandal et al. 2015), etc.

In the current implementation of FMS, relations among microbes were pre-computed from a reference database by their phylogeny of amplified genes (e.g. 16S rRNA) and inferred metabolic functions of corresponding whole genomes, which ensures the comprehensiveness as well as the efficiency for flexible feature extraction. Such preprocessed reference can also be easily reproduced using NCBI RefSeq (O'Leary et al. 2016), SILVA (Christian et al. 2013), RDP (Cole et al. 2014), or other widely used databases for versatile sequence and profile types (e.g. species, OTU, ASV, etc.). This strategy keeps the runtime complexity of Flex Meta-Storms on the same level as other phylogeny-based distance metrics (e.g. Fast UniFrac or Meta-Storms). Meanwhile, it also requires the compositions of all microbiomes are picked from definite references, yet not compatible to *de novo* sequence processing that lacks *a prior* information on community members. On the other side, the marker-based distances may also exhibit limitation in dealing with outliers, where RPCA is advantageous (Cameron et al. 2022).

## 4 Materials and methods

### 4.1 Precomputing of quantitative relations among microbes

To quickly and accurately locate the approximate markers of given exact markers in a microbiome, we precomputed the quantitative relations among microbes in the Greengenes database (v13-8) (DeSantis et al. 2006). Basically, for each OTU (operational taxonomy unit), we trace its approximate neighbors (AN) and the corresponding distances using full-length 16S rRNA gene sequence similarity, taxonomy annotation, and function profile hierarchy. From taxonomy and phylogeny aspects, we used VSEARCH (Torbjørn et al. 2016) to perform pairwise sequence alignment (vsearch—usearch_global) and parse out the top 0.1% sequence similarity threshold *t_s_* (0.92). Then for OTU *i*, we can select its phylogeny neighbors (PN) with both high sequence similarity and identical taxonomy annotation by Equation (1):


(1) $$\mathrm{PN}\;\left(i\right)=\;\forall j\;\in \left\{\left({\mathrm{similarity}}_{s}\;\left(i,\;j\right)\geq t_{s}\right)\;\&\&\left(\mathrm{taxon}\;\left(i\right)=\mathrm{taxon}\left(j\right)\right)\right\}.$$

Similarly, using the KEGG (Minoru et al. 2016) function profiles predicted from PICRUSt 2 (Douglas et al. 2020), we employed Hierarchical Meta-Storms (HMS) algorithm (Zhang et al. 2021) to calculate the functional distance threshold *d_f_* (0.11) and screen functional neighbors (FN) as Equation (2):


(2) $$\mathrm{FN}\;\left(i\right)=\;\forall j\;\in \left\{\mathrm{HMS}\;\left(i,\;j\right)\leq d_{f}\right\}.$$

Finally, the candidate approximate neighbors of OTU *i* can be taken out by the intersection set of PN and FN as Equation (3), which ensured they shared the close taxonomic, phylogenetic, and functional features.


(3) $$\mathrm{AN}\;\left(i\right)=\;\mathrm{PN}\left(i\right)\cap \mathrm{FN}\left(i\right).$$

### 4.2 Bio-marker selection and flexible member extraction

To highlight the advantage from FMS algorithm rather than superior bio-marker selection strategies, we only used the Wilcoxon rank sum test to choose the uneven distributed microbes between different groups (*P*-value < 0.01) as exact markers, and then deduce the approximate markers from their neighbors. Alternatively, exact markers can also be manually assigned as any microbes of interest (Supplementary Fig. S1A). Considering the approximate markers also dilute the original exact markers, here, we developed a flexible extraction method for balance. In a microbiome, a single approximate marker *j* (with relative abundance *Abd_j_*) can be indexed from multiple exact markers. Then its contribution to the local alignment *Abd_j_’* was weight by the sequence similarity to the nearest exact marker as Equation (4). Hence, the exact and approximate markers are merged as the target member set *T* for local alignment (Supplementary Fig. S1B).


(4) Abd'j=maxj∈PN(i)⁡similaritys(i,j) ×Abdj.

### 4.3 Normalized phylogenetic distance of microbial fractions

Based on the target members from the microbiomes, the FMS algorithm calculates the phylogeny distance (Su et al. 2012) of sample pairs with normalization (Supplementary Fig. S1C). The target members of a sample pair are first mapped to leaf nodes of the common binary phylogenetic tree, and then the distance on each branch is calculated recursively from the leaf nodes to the root. Suppose that for a target member *sp* (a tip node in the phylogenetic tree), its relative abundances in two samples are sp. S1 and sp. S2, respectively. We define *Con(sp)* as the consistency score of a single species in Equation (5):


(5) $$\mathrm{Con}\left(sp\right)=\min\left(sp.S1,sp.S2\right),\;sp\in T.$$

And for an internal node *sp’* of the phylogeny tree with two children of *sp_i* and *sp_j*, its consistency score can be extended from Equations (5) to (6):


(6) $$\mathrm{Con}\left(sp^{'}\right)=\min\left(\left|sp\_i.S1-sp\_i.S2|\times (1-d_{1}),|sp\_j.S1-sp\_j.S2|\times (1-d_{2}\right)\right).$$

Here, *d_i_* represents the distance of species *sp_i* to its ancestor node. We recursively process all internal nodes in the phylogenetic tree in this way and obtain the overall consistency score of the target members at the root of the tree. Since the target member set *T* is only a fragment of the entire community, the local alignment distance is then normalized by the sum of the relative abundance of *T* as Equation (7).


(7) $${\mathrm{Dist}}_{\mathrm{local}}\left(S1,\;S2\right)=\frac{2\times (1-\mathrm{Con}(\mathrm{root}))}{\sum_{i\in T}sp\_i.S1+\;\sum_{i\in T}sp\_i.S2}.$$

### 4.4 Microbiome datasets, sequence preprocessing, and statistical analysis

Microbiome datasets used in this work and their information are listed in Table 1. The original sequences were preprocessed by Parallel-Meta Suite (Chen et al. 2022), including chimera removal, pair-end merging, and ASV denoising. OTUs were then picked against Greengenes database (v13-8) with sequence similarity of 0.99, and relative abundance of microbes was normalized and corrected by 16S rRNA gene copy numbers. The phylogenetic tree of OTUs was pre-built by Greengenes using FastTree (Price et al. 2009). PERMANOVA test, ANOSIM test, and multivariate dispersion test were performed using the “vegan” package (Dixon 2003) of CRAN-R (R Core Team 2013). Number of permutations was set as 999, and threshold of significance for *P*-value was set as 0.01.

### 4.5 Code implementation and parallel computing

Flex Meta-Storms algorithm is implemented in C++ with OpenMP-based parallel computing. It contains a preprocessed reference based on Greengenes database (v13-8) (DeSantis et al. 2006) for fast and flexible target member extraction. The software is compatible with operating systems of Linux, Mac, and Windows Subsystem Linux. Taking microbiome samples’ features table [e.g. profiling results from Parallel-Meta Suite (Chen et al. 2022) or QIIME2 (Bolyen et al. 2019)] and exact markers as input, FMS calculates the “local alignment” distances as output. The input exact markers could be provided in two ways (i) manually assigned by users with markers of interest or (ii) automatically selected by FMS package using rank-sum test [Wilcoxon test for two groups, Kruskal test for three or more groups, implemented by CRAN-R (R Core Team 2013)].

## Supplementary Material

btad148_Supplementary_Data

## Data Availability

The software packages and pre-processed datasets are available at GitHub (https://github.com/qdu-bioinfo/flex-meta-storms).
