# Supplementary material for: Flex Meta-Storms elucidates the microbiome local beta-diversity under specific phenotypes
Source: Bioinformatics. 2023 Mar 22;39(4):btad148. doi: 10.1093/bioinformatics/btad148 (PMC10082668; doi:10.1093/bioinformatics/btad148)
Supplement: btad148_Supplementary_Data [file btad148_supplementary_data.docx]

**Supplementary materials for “Flex Meta-Storms elucidates the microbiome local beta-diversity under specific phenotypes”**

**Supplementary Figures**

**
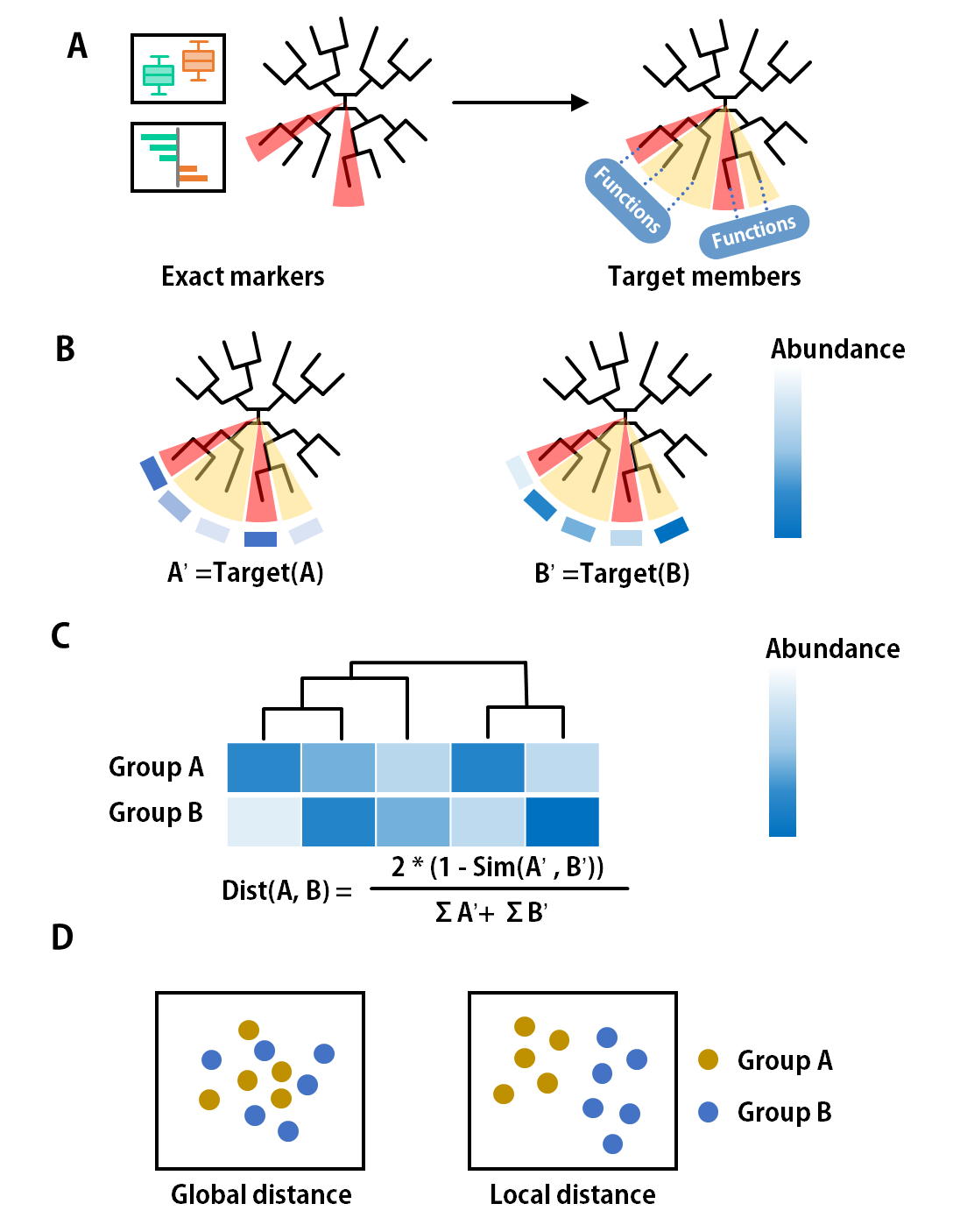
**

**Figure S1. The overall procedure of Flex Meta-Storms.** (**A**) Exact markers can either be performed by biomarker selection tools, or manually assigned by users, then target members were flexibly identified based on phylogenetic and function relations of microbes. (**B**) Extraction of target members. (**C**) Normalized phylogenetic distance of target members. (**D**) The local distances of FMS reveal the hidden beta-diversity pattern missed by global distances.


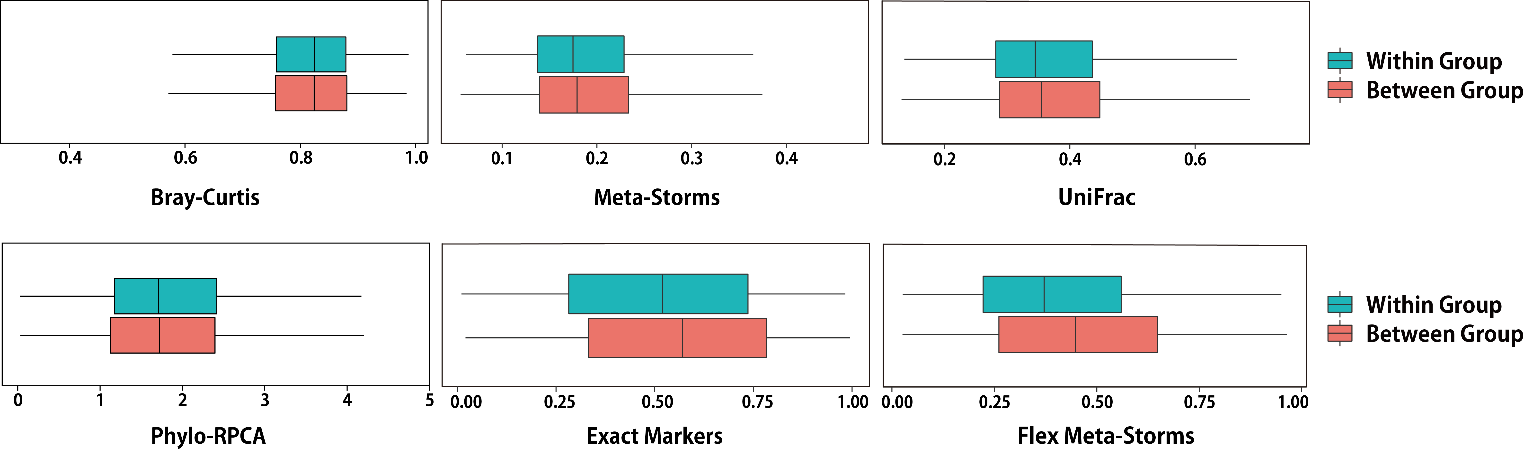


**Figure S2. Between-group and within-group distances for six measures of ASD samples.**


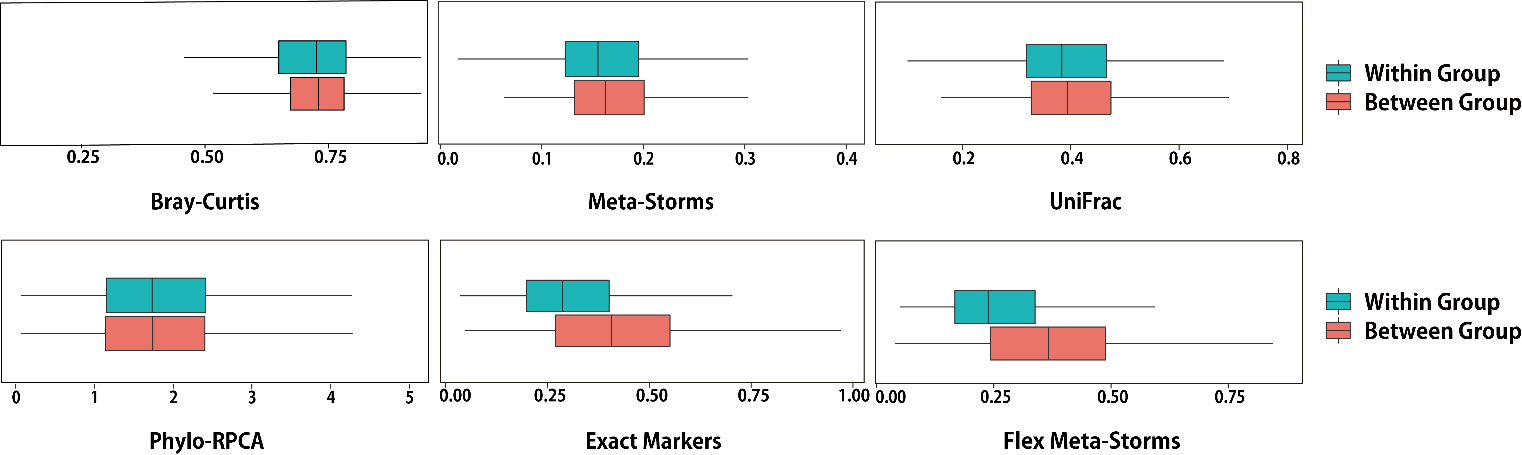


**Figure S3. Between-group and within-group distances for six measures of CRC samples.**

**Supplementary Tables**

**Table S1. Results of ANOSIM test between different status.**

(**A**) Real dataset I

| Distance metrices | Bray-Curtis | Meta-Storms | UniFrac | Phylo-RPCA | Exact markers | FMS |
| --- | --- | --- | --- | --- | --- | --- |
| *R* | 0.003318 | 0.02157 | 0.03557 | -0.007438 | 0.08669 | 0.1532 |
| *p*-value | 0.325 | 0.06 | 0.015 | 0.63 | 0.002 | 0.001 |

(**B**) Real dataset II

| Distance metrices | Bray-Curtis | Meta-Storms | UniFrac | Phylo-RPCA | Exact markers | FMS |
| --- | --- | --- | --- | --- | --- | --- |
| *R* | 0.05022 | 0.09067 | 0.05835 | 0.0006747 | 0.3351 | 0.4119 |
| *p*-value | 0.002 | 0.003 | 0.003 | 0.367 | 0.001 | 0.001 |

**Table S2. Results of** **multivariate dispersion test between different status.**

(**A**) Real dataset I

| Distance metrices | Bray-Curtis | Meta-Storms | UniFrac | Phylo-RPCA | Exact markers | FMS |
| --- | --- | --- | --- | --- | --- | --- |
| *p*-value | 0.204 | 0.166 | 0.716 | 0.272 | 0.52 | 0.955 |

(**B**) Real dataset II

| Distance metrices | Bray-Curtis | Meta-Storms | UniFrac | Phylo-RPCA | Exact markers | FMS |
| --- | --- | --- | --- | --- | --- | --- |
| *p*-value | 0.002 | 0.001 | 0.023 | 0.181 | 0.897 | 0.055 |
